# Establishment of cancer cell line originating from a patient with high-grade serous ovarian carcinoma

**DOI:** 10.2144/fsoa-2023-0025

**Published:** 2023-07-29

**Authors:** Nguyen Thuy Linh, Ngo Thu Hang, Bui Khac Cuong, Dang Thuy Linh, Nham Thi Phuong Linh, Do Nguyen-Van, Tran Ngoc Dzung, Can Van Mao, Dang Thanh Chung, Le Tri Chinh, Nguyen Phu Hung, Hoang Van Tong, Nguyen Linh Toan

**Affiliations:** 1 Department of Pathophysiology, Vietnam Military Medical University, Hanoi, Vietnam; 2 Department of Pathology, Military Hospital 103, Vietnam Military Medical University, Hanoi, Vietnam; 3 Department of Pathology, Hanoi Medical University Hanoi, Vietnam; 4 Laboratory Animal Research Center, Vietnam Military Medical University, Hanoi, Vietnam; 5 Department of Pathophysiology & Immunology, Hanoi Medical University, Hanoi, Vietnam; 6 Department of Gynaecology Surgery, Vietnam National Cancer Hospital, Hanoi, Vietnam; 7 Faculty of Biotechnology, Thai Nguyen University of Sciences, Thai Nguyen, Vietnam; 8 Institute of Biomedicine & Pharmacy, Vietnam Military Medical University, Hanoi, Vietnam

**Keywords:** CD46, cell line, high-grade serous carcinoma, immunohistochemistry, ovarian cancer

## Abstract

**Aim:**

Ovarian cancer is a serious malignancy with high prevalence and mortality.

**Methods:**

We isolated and characterized an ovarian high-grade serous cancer cell line (M4) from a tumor of a Vietnamese patient with ovarian carcinoma.

**Results:**

The M4 cancer cell line showed good proliferation and stability in culture. Morphologically, the M4 cells showed similar characteristics to tumor cells such as a polyhedral shape, large irregular nuclei, high nuclear/cytoplasmic ratio, high nuclear density and expressing cancer markers like CA125, p53 and Ki67 markers.

**Conclusion:**

We have successfully isolated and characterized the M4 cell line from a Vietnamese patient with ovarian carcinoma.

Ovarian cancer is a common malignancy in women with incidence and mortality of 3.4% in 9.2 million new cases and 4.7% in 4.4 million cancer deaths, respectively [1]. Approximately 90% of ovarian cancers are of the epithelial cell type and encompass multiple histological types, each characterized by specific molecular alterations, clinical features and treatment outcomes. The remaining 10% consists of non-epithelial ovarian cancers, predominantly germ cell tumors, sex cord-stromal tumors, and a few exceptionally rare tumors such as small cell carcinomas [2]. If ovarian cancer is diagnosed at an early stage, when the tumor is localized within the ovary, 90% of patients can survive for 5 years. However, if the patient is diagnosed at an advanced stage, the 5-year survival rate drops significantly to only 29% [3]. Among the different forms of ovarian carcinoma, high-grade serous ovarian cancer has the highest prevalence and mortality, ranging from more than 20% to over 60% and mortality from about 70–80% [4,5]. In terms of prognosis, high-grade serous carcinoma is a poor prognosis with a 3-year survival rate of 67.6% [6]. The high prevalence, mortality and poor prognosis of high-grade serous ovarian cancer are mainly due to the difficulty of early diagnosis, the tendency to recurrence, and the resistance to chemotherapy and radiotherapy [5,7]. *BRCA1* and *BRCA2* germline mutations are the most potent recognized genetic risk factors for epithelial ovarian cancer and are detected in 6–15% of women diagnosed with this disease. Epithelial ovarian cancer patients with *BRCA1/2* mutations exhibit a superior response to platinum-based chemotherapies compared with those without *BRCA1/2* mutations, resulting in improved survival rates, despite the disease being diagnosed at an advanced stage and higher grade [8].

Morphological and molecular genetic studies have revealed that ovarian cancer is a heterogeneous lesion with many different types of carcinomas, each with a distinct phylogenetic origin, differentiation and progression [9,10]. The abnormalities associated with the malignant phenotype and the mechanism of ovarian cancer development remain largely unknown. Furthermore, *in vitro* studies face numerous challenges due to the limited number of ovarian cancer cell lines and the heterogeneity of the cell population derived from tumors or peritoneal fluids. Therefore, it is essential to isolate and propagate tumor cells from patients to establish ovarian cancer cell lines for genetic and cancer pathogenesis studies and to evaluate the effectiveness of treatments [11].

This study aimed to isolate and proliferate an ovarian serous cancer cell line from tumor lesions of a Vietnamese ovarian cancer patient to provide material sources for *in vitro* and *in vivo* research of ovarian cancer.

## Methods

### Collection & characterization of the primary tumor

Tumor samples were obtained from a 54-year-old Vietnamese patient who was diagnosed with bilateral ovarian cancer FIGO IIIc and underwent hysterectomy and bilateral salpingo-oophorectomy in March 2020. Morphological, histopathological and immunohistochemical characteristics of the primary tumor of the ovarian cancer patient were analysed by pathologists. The use of the ovarian cancer patient information and tumor samples was approved by the medical research ethics committee of Vietnam Military Medical University, signed on 26 March 2020 and with number 125 GCN-HĐĐĐNCYSH-ĐHYHN of Hanoi Medical University signed on 15 July 2020.

### Ovarian cancer cell isolation

The method for isolating cancer cells was adapted from a previous study [12]. Briefly, ovarian cancer specimens were resected during oophorectomy, stored in sterile vials, and transported to the cell culture laboratory of the Department of Pathophysiology, Vietnam Military Medical University (VMMU, Vietnam). Ovarian cancer cell isolation was performed in a class II biological safety cabinet, and the resulting cell line was named the M4 ovarian cancer cell line (M4). We used 1 cm^3^ sample piece from the tumor of the ovarian cancer patient. The sample piece was washed with 1× PBS (*phosphate-buffered saline*), minced into small pieces with a sharp blade in a sterile petri dish and gently manipulated to avoid cell damage. The specimens were dissolved into 2 ml of RPMI-1640 supplemented with 10% fetal bovine serum (FBS), 100 U/ml penicillin and 100 μg/ml streptomycin (Sigma Aldrich, USA). The resulting cell suspension was separated into three cell culture flasks (T25 flask) with 5.0 ml of cell culture medium, with 250, 500 and 1000 μl of suspension in each flask, respectively. The cell suspensions were cultured in an incubator at 37°C, with 5.0% CO_2_.

### Characterization of cell growth

The M4 ovarian cancer cells isolated from the patient were cultured and examined by a contrast microscope after 24 h of culture. Once the cells adhered to the flask bottom, the culture medium was removed, and the cancer cells were washed twice with 1× PBS. Fresh culture medium (5.0 ml) was added to the cell culture flask, and the cells were continued to be cultured in the incubator at 37°C with 5.0% CO_2_. The growth of cultured cells was monitored using the Countess II FL (Invitrogen-Thermo Fisher Scientific, MA, USA), and the culture medium was changed every 2 days when the cells reached 80% of the culture flask area. The culture cancer cells were harvested using 1× Trypsin-EDTA and then determined using the Neubauer counter and optical microscope. A standard density of 10^7^ cells per ml was prepared for further experiments.

### Animal experiments

Male BALB/c nude mice, aged 6–8 weeks, were purchased from BioLASCO (Taipei City, Taiwan) and maintained in a pathogen-free environment in accordance with Animal Center Guidelines. All experimental procedures involving animals were conducted following the Institutional Animal Care and Use Committee of the VMMU, Vietnam, and approved by the Ethical Committee. Carbon dioxide inhalation was used to perform all mouse surgeries and euthanasia, and all efforts were made to minimize suffering. To establish BALB/c nude mice bearing M4 human ovarian cancer tumors by xenograft, we inoculated 15 mice with 0.5, 1.0, or 2.0 plus 10^6^ M4 cells in 50 μl FBS on the right rear flanks of BALB/c nude mice, depending on the number of M4 cells. The mice were divided into three groups, each with five mice. One week after inoculation, the mice were checked twice a week for tumor formation, and the size of the tumors was measured and recorded. When the tumors reached 5–10 mm in diameter after 28 days, they were removed from the mice and analyzed for morphology, histopathology and immunohistochemistry. Additionally, human cancer tumors removed from BALB/c nude mice bearing M4 ovarian carcinoma cells were cultured under similar conditions, and their growth characteristics were evaluated.

### Morphology & immunohistochemical stainings

The cultured cells were checked under a contrast microscope. The M4 cell samples isolated from patients were collected from the culture bottles and centrifuged. The cell residues were then washed three-times with 10% FPS solution and a small fraction of the ovarian cancer cell deposits were smeared onto stained glass slides. The fixed cells were left to dry naturally and then stained with Leica's PAP dye following the manufacturer's staining steps. Thrombin and human plasma were added to the majority of the cell residues to form a solid cell mass, which was then fixed with 10% neutral formalin for 4 h before being processed by an automatic sample transfer machine. The mass was then embedded in paraffin and thinly sliced into 3 μm sections before being stained with H.E and immunohistochemistry. Similarly, tumor samples from patients and tumor tissues from nude mice were fixed with 10% neutral formalin for 4 h using the automatic sample transfer machine, embedded in paraffin, and thinly sliced into 3 μm sections before being stained with H.E and analyzed histologically. The slides were stained with PAP (Papanicolaou staining) and H&E (Hematoxylin and Eosin) for histopathological assessment and performing immunohistochemical staining for expression biomarkers including WT1 (Wilson tumor-1), CA125 (cancer antigen 125), Ki67 and p53 proteins using monoclonal antibodies (Leica Biosystems, Wetzlar, Germany).

### Chromosomal analysis

The M4 ovarian cancer cells, which had been cultured for five passages, and the cells from BALB/c nude mice tumors were cultured in 25 cm^2^ flasks and treated with 100 μl of Colcemid (10 μg/ml) 6 h before harvesting. The cells were then centrifuged at 1500 cycles per minute for 10 min to remove fluid and collect cell deposits, and they were placed in a 0.56% KCl solution for 15 min at 37°C. Afterward, the cell residues were centrifuged and fixed with Carnoy's solution (3 parts methanol to 1 part acetic acid). The small cell residue solution was aspirated onto a clean slide and allowed to dry naturally at room temperature. After stabilization, the specimen was G-band stained, and cell samples were taken, 10 chromosome clusters were photographed, and chromosome sets were analyzed using Smartype V software.

### Flow cytometric analysis

Flow cytometry analysis was performed to examine the expression of the CD46 receptor in M4 ovarian cancer cells cultured from the primary tumor of the patient and the secondary tumor of BALB/c nude mice. The cells were cultured at a density of 10^5^ cells/ml and treated with anti-Hu CD46 monoclonal antibody (Invitrogen-Thermo Fisher Scientific). The analysis was carried out using the FACSlyric-BD system (NJ, USA) to determine the ratio of ovarian cancer cells positive for CD46 expression.

### The secondary tumor cell structure under transmission electron microscopy

Subcutaneous xenografts of M4 human ovarian cancer cells were established in BALB/c nude mice. The resulting secondary tumors were collected, washed, and cut into small 1 mm^3^ blocks. The blocks were then fixed in a solution of 3.0% glutaraldehyde and 0.2 M cacodylate (pH = 7.3) at a ratio of 1:10 (sample volume to solution volume) for 1 h at room temperature. After three washes with 0.1 M cacodylate solution for 5 min each, the samples were fixed with 1% OsO4 in 0.1 M cacodylate solution (pH = 7.3) for 1 h at room temperature, followed by two additional 5-min washes with 0.1 M cacodylate solution. The samples were then dehydrated with increasing concentrations of alcohol (50, 70, 80, 90 and 100%), transferred to a propylene oxide solution for 15 min, and then to a 1:1 mixture of propylene oxide and Epon for 60 min. The samples were finally molded with Epon in gelatin at 60°C for 48 h and cut into 50 nm-thick sections. The sections were stained with 5.0% uranyl acetate for 10 min, washed twice with distilled water, stained with lead citrate for 5 min, and washed twice with distilled water. Samples were observed under an electron microscope (JEM 1400, JEOL, Japan).

## Results

### Macroscopic, histopathological & immunohistochemical characteristics of primary tumor from the Vietnamese ovarian cancer patient

**Figure 1.**
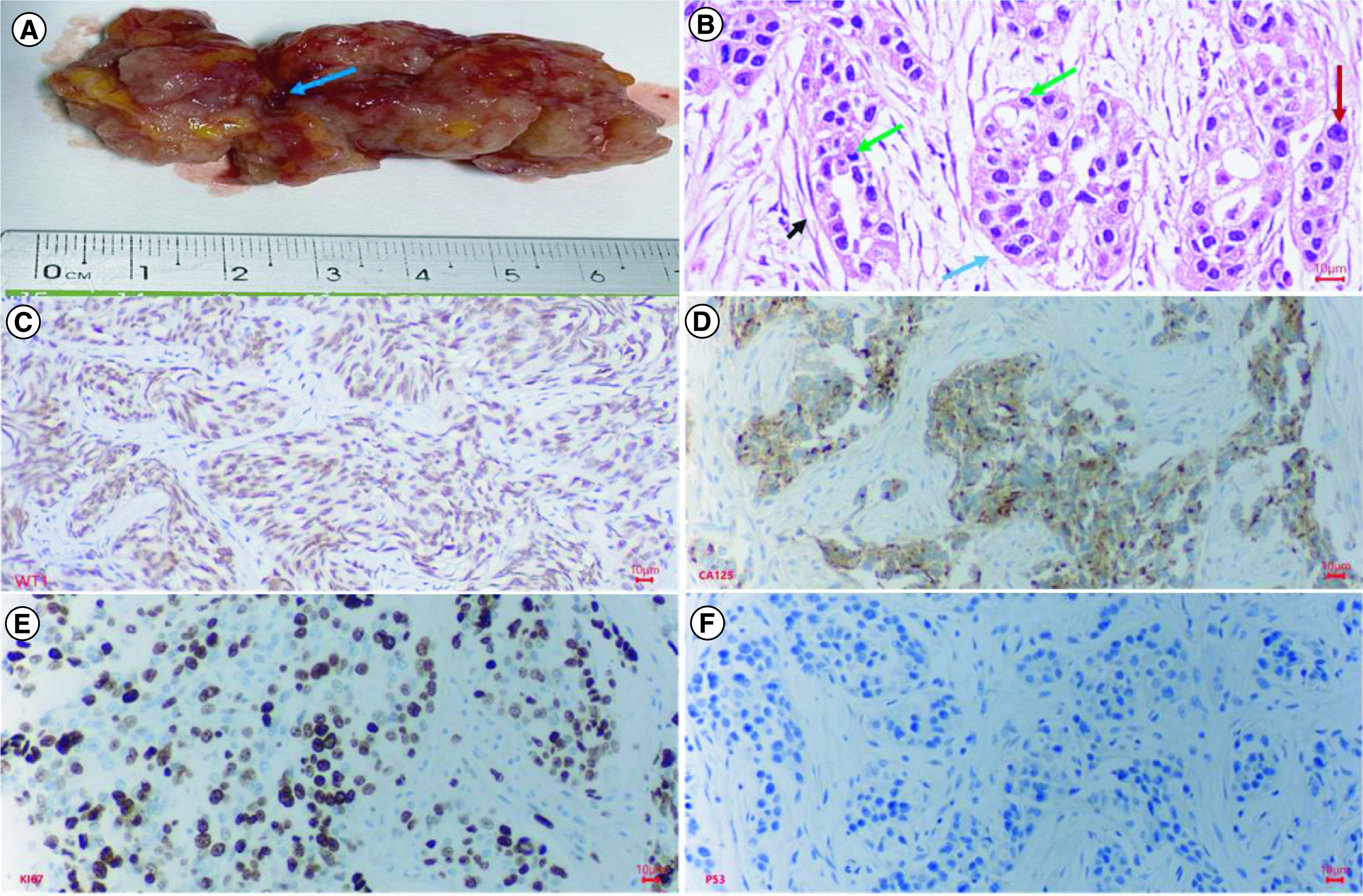
Macroscopic, histopathological and immunohistochemical characteristics of primary tumor of the ovarian cancer patient. Histopathology and immunohistochemistry of ovarian cancer. **(A)** This is a section of the tumor that shows solid areas ranging from tan to white, with minimal necrosis and hemorrhage (blue arrow). The central part of the tumor was taken for histopathology. **(B)** Glandular and cribriform patterns (black and blue arrow). The tumor consists of columnar to cuboidal cells with eosinophilic cytoplasm. Prominent nucleolus (red arrow), often large and eosinophilic has to be seen. A high mitotic index is ≥12 mitotic figures per 10 high power fields, which is often atypical (green arrow). **(C)** Malignant cells show nuclear staining with WT1; **(D)** Malignant cells show strong and diffuse membranous staining with CA125; **(E)** Malignant cells show strong and diffuse nuclear staining with Ki67(red arrow), accounted 70%; **(F)** Malignant cells show a complete absence of staining with p53, corresponding to loss of function mutation that results in a truncated protein that is not detected.

The tumor was opaque white and brownish, lacked a capsule, and histopathologically was identified as a high-grade serous carcinoma (Figure 1). The tumor invaded the uterine wall, appendix, peritoneal metastasis, and omentum metastasis, but there was no abdominal lymph node metastasis. The preoperative serum tumor markers were a CA125 level of 849 U/ml (normal concentration <35 U/ml) and an HE4 level of 1427 pmol/l (normal concentration <76.2 pmol/l). Immunohistochemistry analysis showed that the tumor expressed WT1, CA123, Ki67 and p53 proteins (Figure 1).

### Morphological characteristics of the M4 ovarian cancer cell line from high-grade serous carcinoma

**Figure 2.**
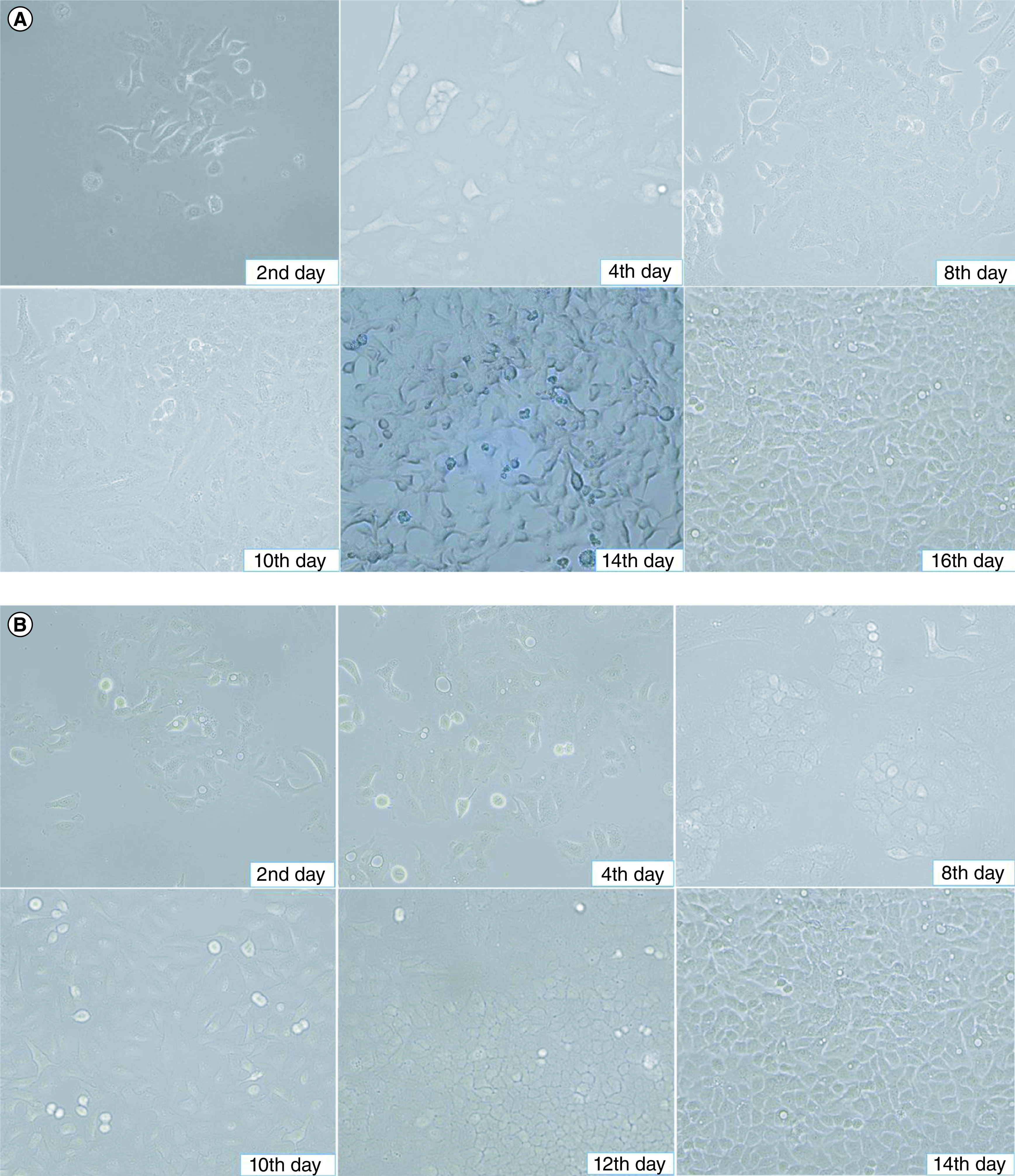
Morphological characteristics of M4 ovarian cancer cells in culture. **(A)** M4 cancer cells in culture on the second day before washing, there were basal tumor cells and reached 10% on the 4th day. On the eighth day, cells adhered to the bottom 40% of the culture area. On the tenth day, cells adhered to the bottom 60% of the culture area. On the fourteenth day, tumor cells had basal epithelial morphology >80% of the culture area. On the sixteenth day after the first sample separation, the tumor cells had a strong proliferative epithelial morphology, attached to the bottom of more than 90% of the culture area. **(B)** BALB/c nude mice's secondary tumor cell in culture. The proliferation speed of tumor cells was similar to that of M4 cells isolated from the patient.

After adding cell suspensions of 250, 500 and 1000 μl to three culture flasks, the flask with 500 μl of cell suspension demonstrated the best growth. After 24 h of culturing, the M4 ovarian cancer cells collected from the patient adhered to the bottom of the culture flask and showed robust proliferation. By day 8, the M4 cancer cells covered 40% of the flask area, and by day 10, 60%. On day 16, they covered over 80% of the flask area. The M4 tumor cells exhibited polygonal shapes, large cytoplasm, nucleoli at the center of the cell, and few spindle cells, as observed under light microscopy (Figure 2).

The secondary M4 ovarian cancer cells collected from BALB/c nude mice were isolated and cultured under similar conditions to the primary M4 ovarian cancer cells from the patient. Contrast microscopy revealed that these secondary M4 cells also adhered to the bottom of the culture flask after 2 to 4 days of culture. After 10 days, the cultured cells had covered 60% of the flask area, and over 80% by day 14 (Figure 2).

### Growth characteristics of the M4 ovarian cancer cell line

**Figure 3.**
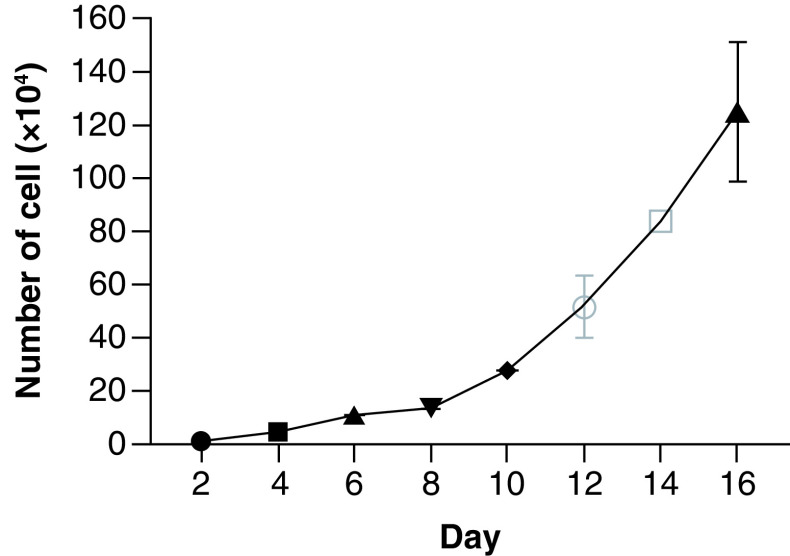
Growth curve of the high-grade serous ovarian cancer cell line.

Cultured M4 cancer cells were counted daily using an automated cell counter (Countess II FL, Invitrogen) until day 16 when the cell count reached 10^6^ cells/ml. Subsequently, the tumor cells were passaged into new T75 flasks with a 1:3 split ratio. This process was repeated for 15 cycles, with each cycle consisting of cell separation and proliferation for 3 months. The tumor cells demonstrated robust proliferation with an average cell doubling rate of 48 h. The cells were harvested using 1× Trypsin-EDTA, stored at -80°C for 24 h, and then transferred to liquid nitrogen at -196°C. These M4 cancer cells were later thawed and re-proliferated, demonstrating stable cell growth (Figure 3).

Tumor cells isolated from immune-deficient mouse tumor tissue bearing M4 human tumours adhered to the bottom of the culture flask, covering over 60% of the surface within 24 h of isolation, and exhibited robust proliferation. The percentage of viable cells reached over 90% after 1 week of isolation and increased to over 95% after 2 weeks. After undergoing four rounds of separation, the tumor cells demonstrated excellent proliferation, with the proliferation rate of the M4–2 cell line doubling approximately every 48 h. The harvested cells were titrated to a concentration of 10^6^ cells/ml, placed in a deep incubator at -80°C for 24 h, and then stored in a liquid nitrogen flask at -196°C for long-term preservation. This preserved cell sample was later retrieved for re-growth. After multiple cycles of proliferation, sample collection, and preservation, all experiments consistently demonstrated the stable proliferation of the tumor cells, with the proliferation rate of the M4-2 cell line doubling approximately every 48 h.

### Immunochemical characteristics of the M4 ovarian cancer cell line

**Figure 4.**
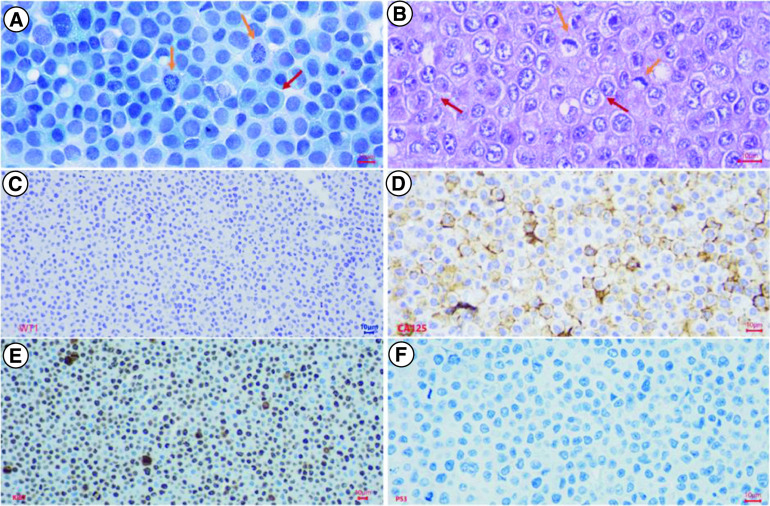
Histopathological and Immunochemical characteristics of M4 ovarian cancer cells in culture. After isolation, the M4 ovarian cancer cells were stained with PAP, H&E, and IHC to express the WT1, CA125, Ki67, and p53 proteins. **(A)** PAP-stained isolated tumor cells showed high cell density, large polygonal epithelial cells with abundant basophilic cytoplasm (red arrow), and a high nuclear/cytoplasmic ratio. Nuclei were round or oval, rough chromatin, mitotic nuclei visible (yellow arrows); **(B)** H&E-stained isolated tumor cells revealed high cell density, epithelial cells with adhesions, polygonal in shape (red arrow), some cytoplasmic had a light cavity. Tumor cells had a high nuclear/cytoplasmic ratio (yellow arrow). Cell nucleus was round or oval, with rough chromatin, revealing many abnormal nuclei (green arrow); **(C)** Immunochemistry staining. Malignant cells didn't reveal nuclear staining with WT1; **(D)** Immunochemistry staining. Malignant cells showed strong and diffuse membranous staining with CA125; **(E)** Immunochemistry staining. Malignant cells expressed strong and diffuse nuclear staining with Ki67, accounting for 80%; **(F)** Immunochemistry staining. Tumor cells revealed a complete absence of staining with p53.

After isolation, the M4 ovarian cancer cells were stained with PAP, revealing unique tumor cells with a polyhedral shape, large irregular nuclei, a high nuclear/cytoplasmic ratio, basophilic nuclei, uncommon nuclear density, and few cytoplasms (Figure 4). These features were similar to the histopathological features of the primary tumor. Immunohistochemical analysis was performed on the isolated M4 ovarian cancer cells to assess the expression of WT1, CA125, Ki67, and p53 proteins. The results did not show the expression of the WT1 marker. However, the expressions of CA125 and p53 proteins were similar to the presentation of the primary tumor, and the Ki67 proliferation index was 80% (Figure 4).

### Morphological & immunohistochemical characteristics of cancer cells isolated from BALB/c nude mice bearing the M4 human ovarian cancer cells

**Figure 5.**
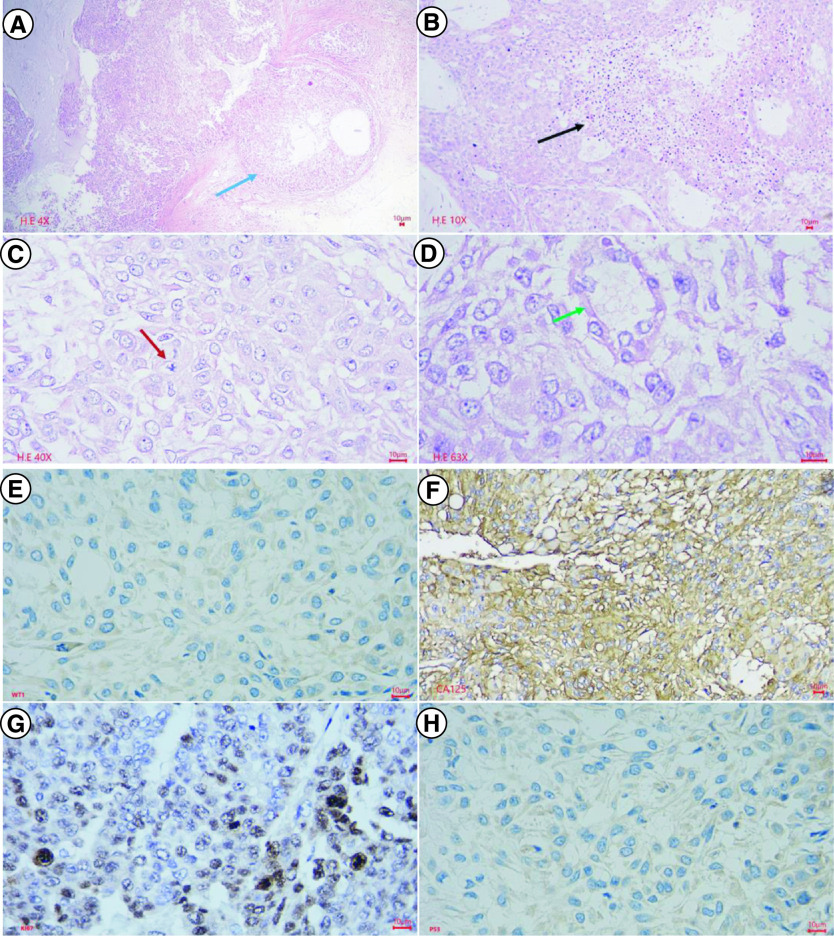
Histopathological characteristics of secondary tumor resected from BALB/c mice. The central part of the primary tumor for histopathology, avoiding the area of gross hemorrhagic necrosis. In Hematoxylin-Eosin staining **(A–D)**, at the low power field, the tumor invades the fibrous capsule (blue arrow), and central necrosis is extensive (black arrow). At the high power field, the tumor shows high cell density, epithelial cells with tight adhesion to each other, in some places similar to glandular or cystic structures (green arrow), polygonal in shape and some cytoplasms with light cavities. High nuclear/cytoplasmic ratio, round or oval nucleus, rough chromatin, and abnormal nuclei mitosis (red arrow). In immunohistochemical staining **(E–H)**, malignant cells showed negative nuclear staining with WT1, strong and diffuse membranous staining with CA125, nuclear staining with Ki67 accounted for 60%, and the complete absence of staining with p53.

To investigate the cancerous characteristics of M4 ovarian cancer cells, we performed xenograft experiments in BALB/c nude mice. After subcutaneous injection of M4 human ovarian cancer cells, tumors appeared in one mouse of group 1 and two mice each of groups 2 and 3 on day 4. On day 10, all five mice in groups 2 and 3 had tumors, while only two mice in group 1 had tumors that remained until day 28. After 28 days, we resected the tumors for morphological and histopathological examinations, which showed that the M4 human ovarian cancer cells isolated from BALB/c nude mice had the same characteristics as the patient's primary M4 ovarian cancer cells. However, the tumors consisted mainly of proliferative epithelial cells, without fibroblasts (Figure 5). Immunohistochemical staining revealed that the secondary M4 ovarian cancer cells isolated from tumors of BALB/c nude mice were similar to the primary M4 ovarian cancer cells isolated from the patient, with no expression of WT1 protein, overexpression and diffuse CA125 protein, complete absence of p53, and a high Ki67 protein proliferation index (60%) (Figure 5).

**Figure 6.**
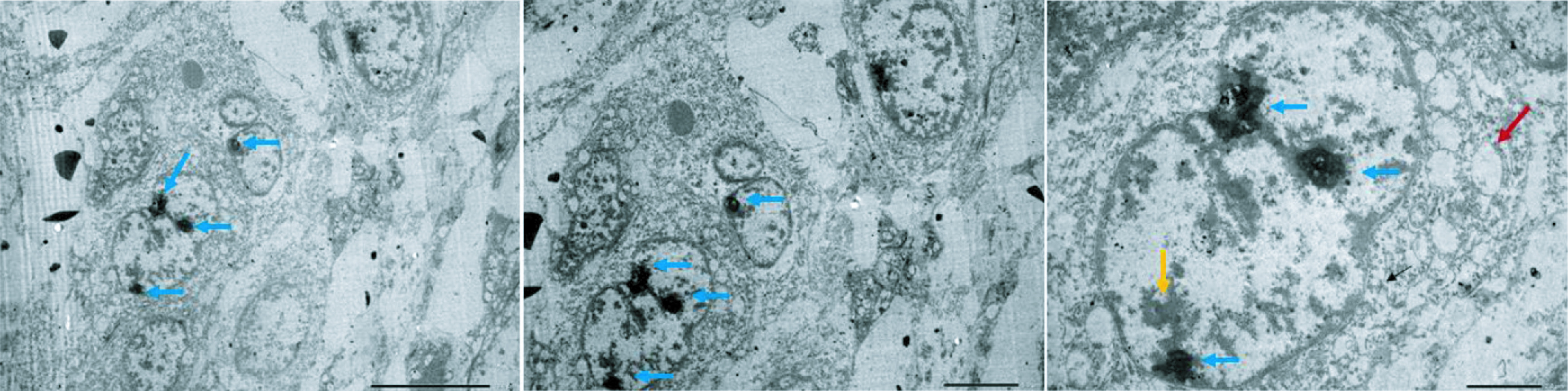
Electron micrograph of the secondary tumors. Electron micrograph showed that tumor cells possessed large size and high nuclear/cytoplasmic ratio. The cells had the unequal thickness of nuclear membranes (yellow arrow), with many nuclei (blue arrow), uneven distribution of chromatin, and vacuoles in the cytoplasm (red arrow).

Electron microscopy analysis revealed further details about the morphology of the M4 ovarian cancer cells. The cells exhibited a large size with a high nuclear/cytoplasmic ratio, indicating their malignant characteristics. The nuclear membranes of the M4 cells were found to be uneven in thickness, and many nuclei were observed. Additionally, the distribution of chromatin and vacuoles in the cytoplasm was found to be irregular, suggesting the abnormality of the cell structure (Figure 6).

### Chromosomal characteristics of the M4 ovarian cancer cell line

**Figure 7.**
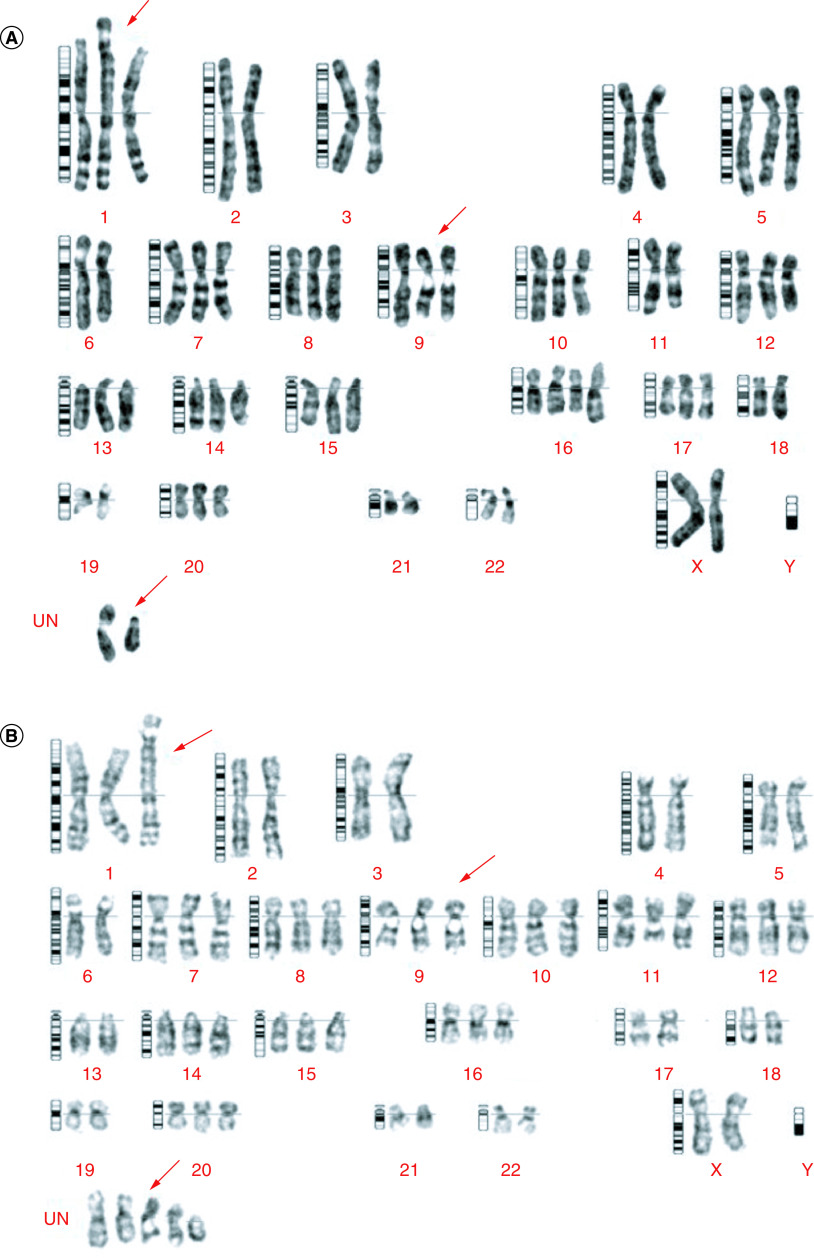
Karyotyping of M4 cancer cell line. Karyotyping was performed on M4 cell samples isolated from both humans and mice. The cells were cultured for 10 days until the tumor growth reached about 80% of the bottom area of the culture plate. **(A)** Karyotyping of a cell line separated directly from the tumor. The figure shows that 62 chromosomes were in metaphase, 11 were trisomy, one was tetrasomy, and translocations occurred between two chromosomes, 1 and 6. Occurrence 2 chromosomes of undetermined type. **(B)** Karyotyping of a cell line from a tumor in mice. The figure shows that 62 chromosomes were in metaphase, 11 were trisomy, five were undetermined type, and translocations occurred between two chromosomes, 1 and 6.

Chromosomal analysis of M4 ovarian cancer cells, isolated from the patient's tumor and BALB/c nude mice bearing the M4 human ovarian cancer cells by xenografting, revealed stable chromosome aberrations on the M4 isolated cell line. In metaphase, we observed 62 chromosomes, of which 11 were trisomy and one was tetrasomy. We also detected a translocation between chromosome 1 and chromosome 6, and two unidentified chromosomes (Figure 7).

### CD46 expression

**Figure 8.**
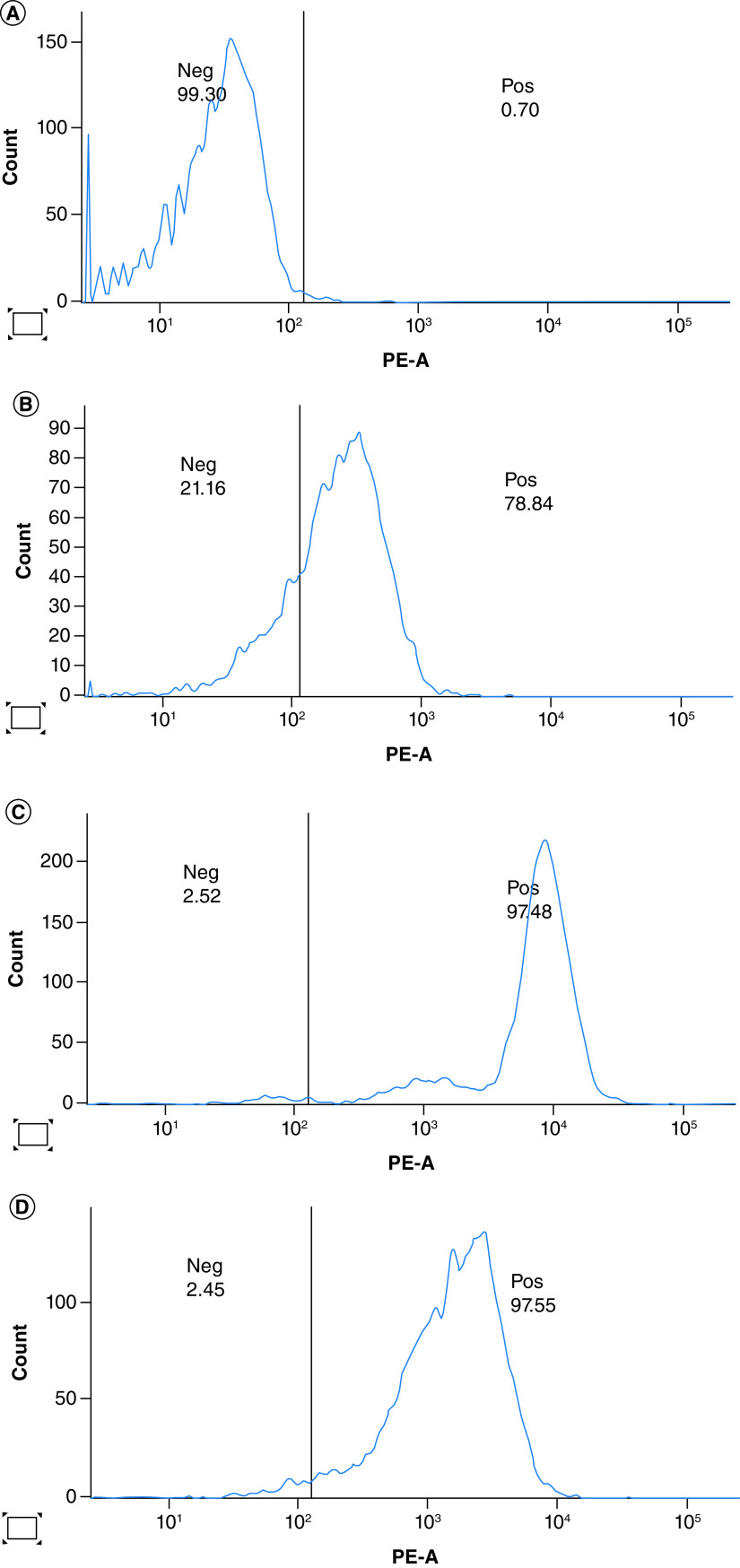
Flow cytometry of CD46 expression of cancer cells. **(A)** No staining CD46; **(B)** The patient's primary tumor cells; **(C)** The M4 cultured cancer cells; **(D)** The secondary tumor cells from BALB/c nude mice.

Twenty high-grade serous carcinoma specimens were obtained from the patient, while 13 samples of M4 ovarian cancer cells were collected during isolation and proliferation from primary ovarian cancer tissue. Additionally, 20 secondary ovarian cancer cells were collected from tumor tissue of BALB/c nude mice bearing the M4 human ovarian cancer cells via xenografting. All M4 cancer cells were stained to determine the expression of the CD46 receptor using flow cytometry analysis. The results indicated strong CD46 expression in primary M4 ovarian cancer cells, cultured M4 ovarian cancer cells, and secondary M4 human ovarian cancer cells isolated from the tumor of BALB/c nude mice at a high and stable rate. The cultured M4 ovarian cancer cells and secondary M4 human ovarian cancer cells from BALB/c nude mice exhibited high homogeneity with a significantly higher CD46 expression ratio compared with the primary M4 ovarian cancer cells (Table 1; Figure 8).

**Table 1.** CD46 expression on M4 ovarian cancer cells.

Tumors	n	The ratio of cells positive with CD46
		Median (25%–75%)
The primary M4 ovarian tumor cells of the patient	20	74.36 (54.785–84.68)
The M4 cultured ovarian cancer cells	13	91.33 (64.145–97.515)
The secondary M4 human ovarian tumor cells from BALB/c nude mice	20	96.353 (91.308–98.316)

## Discussion

Human cell sources for research purposes vary widely, ranging from pluripotent stem cells to cancer cells, and all can be cultured and propagated for various applications. In this study, primary ovarian cancer cells were utilized for proliferation and implantation in animal experiments. This M4 cancer cell line is characterized by relatively similar tumor cells, polyhedral shape, large irregular nuclei, high nuclear/cytoplasmic ratio, basophilic nuclei, high uncommon nuclear density, and few cytoplasms. The M4 ovarian cancer cells were successfully implanted in BALB/c nude mice and grew into tumors with high histopathological similarities to the primary M4 ovarian tumor of the patient.

Previously, three cell lines of ovarian carcinoma (TOV-1946, TOV-2223 G and OV-1946) have been established from the patient's tumor and the peritoneal fluid. Similarly, the OMC-3, HCH-1, and NOMH-1 cell lines were taken from a tumor of patients with ovarian cancer [12–15]. Zhuangyu Pan (2012) established the OVCA 12 cell line from serous ovarian carcinoma [16]. Fernanda Silva (2022) successfully isolated the serous ovarian cancer cell line IPO43 from the peritoneal fluid of high-grade serous ovarian cancer treated with adjuvant chemotherapy [17]. The OVCAR-3 ovarian cancer cell line that was basal cell type had been cultured continuously for 10 months and developed stably after 20-times of separation with a cell doubling rate of 48 h [18]. The OMC-3 cell line obtained from a patient with serous adenocarcinoma grew well for 65 months and underwent more than 50-times of subcultures; the HCH-1 cancer cell line grew well for 230 months and over 50-times of subcultures; the NOMH-1 cell line developed stably over 232 months and more than 50-times of subcultures [12–14]. Successfully cultured cell lines from ovarian carcinoma in studies worldwide have epithelial morphology such as cell lines of TOV-1946, TOV-2223G, and OV-1946. OMC-3, HCH-1, NOMH-1 and OVCAR-3 [12–15,18].

In this study, stable chromosome aberrations observed were mainly hypertriploid abnormalities and translocations. Some serous ovarian cancer cell lines such as TOV-2223, TOV-1946, OV-194 and IPO436 have also shown chromosomal abnormalities [15,17]. Yamada's study on the NOMH-1 cell line from endometrioid adenocarcinoma also showed some hypertriploid aberrations with trisomy and tetrasomy, 6q-, and 8p+, as well as some unidentified chromosomes [13]. Chromosome aberrations have also been observed in primary ovarian cancers with changes in both numeration and structure [19]. Genetic modifications in DNA molecules, such as point mutations, deletions, additions, or transpositions of nucleotides, as well as deletions, additions, or translocations of chromosome aberrations, may be related to oncogene activation and anti-oncogene inactivation [16]. While p53 mutations were not tested in our study, many studies have utilized immunohistochemistry as a substitute for molecular biology testing to detect p53 mutations. These studies identified p53 protein overexpression or non-expression in the tumor cell nucleus, or p53 protein overexpression in the tumor cell cytoplasm, as potential indicators of p53 gene mutation on the immunohistochemical phenotype [20]. A previous study suggested that the use of genomically-informed cell line models for all tumor types can bridge the gap between cell lines and tumors. This can help optimize the selection of cell lines as tumor models for cancer research [21].

The histopathology of high-grade serous carcinoma reveals that the stromal component has a lower density compared with the tumor cells. The cancer cells demonstrate a high mitotic index, and the tumor structure is denser than the cystic part. These histological features suggest that high-grade serous carcinoma is more accessible for isolation than other types of ovarian cancer, such as the predominantly mucinous cyst type with abundant extracellular mucin, where the cancer cell density is typically low, and the proliferation index is also low. Endometrial and neuroendocrine cancer types often have a solid form with a high density of cell proliferation, but their cell adhesion remains stable, making it difficult to isolate cancer cells. Different histopathological types of ovarian carcinoma, including serous carcinoma, clear cell carcinoma, mucinous carcinoma, and endometrial carcinoma, have been isolated into commercial products and have been widely published [12–14,18]. Immunohistochemical staining of the M4 ovarian cancer cell line revealed similar expression of CA125, p53, and Ki67 proteins in the primary M4 ovarian tumor. However, WT1 is not expressed in the M4 cell line (primary tumor with WT1 expression). These results were also observed in secondary M4 ovarian tumors implanted in BALB/c nude mice.

CD46 is a membrane protein that inhibits complement activation by host cells, occurring on the surface of many epithelial, endothelial and hematopoietic cells, but absent in red blood cells. The expression of CD46 has been demonstrated in various tumors and cell lines derived from tumors. The expression of the CD46 receptor on cancer cells was more pronounced than in surrounding healthy tissues. The increased expression of CD46 on tumor cells suggests the emergence of a mechanism to protect cancer cells from the body's immune response. Ovarian cancer is one of the tumors expressing CD46 protein on the tumor cell surface. The CD46 receptor expression is associated with poor prognosis as they predict early disease recurrence [22]. CD46 expression on the surface of cancer cells was not associated with a histopathological subtype of ovarian cancer, tumor cell differentiation, and response to treatment [22]. An earlier study demonstrated that 60% of primary ovarian tumors have CD46 receptor expression on the tumor cell membrane [22]. In our study, both primary and secondary M4 ovarian cancer cells isolated and cultured from the patient's tumor and BALB/c nude mice bearing the M4 cell line exhibited high and stable CD46 expression. The expression of CD46 was significantly more homogeneous than that of the patient's primary cancer cells. These findings suggest that our M4 cell line possesses the characteristics of cancerous cells.

Although this study is the first to isolate and characterize an ovarian cancer cell line from a Vietnamese patient, it still has some limitations. To demonstrate the growth and stability of the cell line, more passages or a longer culture period should be carried out. Additionally, genetic profiling, such as STR analysis, gene expression, mutation and karyotyping analysis of different passage stages should be conducted to confirm the stability of the established ovarian cell line.

## Conclusion

A cell line of high-grade serous ovarian cancer (M4) has been successfully isolated and characterized from a Vietnamese patient with ovarian carcinoma. The establishment of the M4 ovarian cancer cell line provides valuable insights for basic research in ovarian cancer, particularly for high-grade serous ovarian cancer. Furthermore, this M4 ovarian cell line from a Vietnamese patient will be used in further studies to gain a better understanding of the molecular mechanisms, pathogenesis, and treatment of serous ovarian cancer.

**Summary points**

- Ovarian cancer is a dangerous malignancy with high prevalence and mortality worldwide, especially high-grade serous ovarian carcinoma.
- This study established an ovarian high-grade serous cancer cell line (M4) from tumor of a Vietnamese patient with ovarian carcinoma.
- An ovarian carcinoma cell line (M4) was isolated from a Vietnamese 54-year-old patient with a high-grade serous ovarian carcinoma tumor.
- The M4 cell line was characterized for morphological, histopathological and immunohistochemical features, tumor markers, and the ability to form tumors in BALB/c nude mice.
- The M4 ovarian cancer cell line has been proliferating well and is stable in the culture condition.
- The M4 cells showed similar characteristics to tumor cells such as polyhedral shape, large irregular nuclei, high nuclear/cytoplasmic ratio, basophilic nuclei, high uncommon nuclear density, and few cytoplasms.
- These cells were transplanted into BALB/c nude mice and formed tumors with similar characteristics to the primary tumor. The M4 ovarian cancer cells expressed CA125, p53, and Ki67 immunohistochemical markers like the primary tumor, except WT1.
- The ovarian high-grade serous cancer cell line (M4) has been successfully isolated and characterized from a Vietnamese patient with ovarian carcinoma.
- The established M4 ovarian cancer cell line from a Vietnamese patient shall help further investigate high-grade serous ovarian cancer.

## References

[1] Sung H, Ferlay J, Siegel RL Global cancer statistics 2020: GLOBOCAN estimates of incidence and mortality worldwide for 36 cancers in 185 countries. CA Cancer J. Clin. 71(3), 209–249 (2021). 33538338 10.3322/caac.21660

[2] Cheung A, Shah S, Parker J Non-Epithelial Ovarian Cancers: How Much Do We Really Know? Int. J. Environ. Res Public Health 19(3), 1106 (2022). 35162125 10.3390/ijerph19031106 PMC8834485

[3] Torre LA, Trabert B, DeSantis CE Ovarian cancer statistics, 2018. CA Cancer J. Clin. 68(4), 284–296 (2018). 29809280 10.3322/caac.21456 PMC6621554

[4] Coburn S, Bray F, Sherman M, Trabert B. International patterns and trends in ovarian cancer incidence, overall and by histologic subtype. Int. J. Cancer 140(11), 2451–2460 (2017). 28257597 10.1002/ijc.30676 PMC5595147

[5] Mei J, Tian H, Huang H-S Cellular models of development of ovarian high-grade serous carcinoma: a review of cell of origin and mechanisms of carcinogenesis. Cell Prolif. 54(5), e13029 (2021). 33768671 10.1111/cpr.13029 PMC8088460

[6] Lee KR, Muto MG. Chapter 25 - The Pathology of Pelvic-Ovarian Epithelial (Epithelial-Stromal) Tumors. In: Diagnostic Gynecologic and Obstetric Pathology (3rd Edition). Howitt Edition BE, Nucci MR, Crum CP, Granter SR, Parast MM, Boyd TK (Eds). Elsevier, PA, 865–948 (2018).

[7] Franier BDL, Thompson M. Early stage detection and screening of ovarian cancer: a research opportunity and significant challenge for biosensor technology. Biosens. Bioelectron. 15(135), 71–81 (2019). 10.1016/j.bios.2019.03.041 31003031

[8] Boussios S, Rassy E, Moschetta M BRCA Mutations in Ovarian and Prostate Cancer: Bench to Bedside. Cancers (Basel) 14(16), 3888 (2022). 36010882 10.3390/cancers14163888 PMC9405840

[9] Stewart C, Ralyea C, Lockwood S. Ovarian Cancer: An Integrated Review. Semin. Oncol. Nurs. 35(2), 151–156 (2019). 30867104 10.1016/j.soncn.2019.02.001

[10] Armstrong DK. Relapsed ovarian cancer: challenges and management strategies for a chronic disease. The Oncologist 7(Suppl. 5), 20–28 (2002). 10.1634/theoncologist.7-suppl_5-20 12324630

[11] Sueblinvong T, Ghebre R, Iizuka Y Establishment, characterization and downstream application of primary ovarian cancer cells derived from solid tumors. PLOS ONE 7(11), e50519 (2012). 23226302 10.1371/journal.pone.0050519 PMC3511542

[12] Yamada T, Hattori K, Satomi H, Okazaki T, Mori H, Hirose Y. Establishment and characterization of a cell line (HCH-1) originating from a human clear cell carcinoma of the ovary. J. Ovarian Res. 9(1), 32 (2016). 27259990 10.1186/s13048-016-0242-y PMC4893251

[13] Yamada T, Kanda T, Mori H, Shimokawa K, Kagawa M, Shibayama Y. Establishment and characterization of a cell line (NOMH-1) originating from a human endometrioid adenocarcinoma of the ovary. J. Ovarian Res. 6(1), 8 (2013). 23379414 10.1186/1757-2215-6-8 PMC3568727

[14] Yamada T, Ueda M, Otsuki Y, Ueki M, Sugimoto O. Establishment and characterization of a cell line (OMC-3) originating from a human mucinous cystadenocarcinoma of the ovary. Gynecol. Oncol. 40(2), 118–128 (1991). 2010102 10.1016/0090-8258(91)90102-b

[15] Ouellet V, Zietarska M, Portelance L Characterization of three new serous epithelial ovarian cancer cell lines. BMC cancer 8, 152 (2008). 18507860 10.1186/1471-2407-8-152 PMC2467432

[16] Pan Z, Hooley J, Smith DH, Young P, Roberts PE, Mather JP. Establishment of human ovarian serous carcinomas cell lines in serum free media. Methods 56(2012), 432–439 (2012). 22445873 10.1016/j.ymeth.2012.03.003

[17] Silva F, Coelho F, Peixoto A Establishment and characterization of a novel ovarian high-grade serous carcinoma cell line – IPO43. Cancer Cell International 27(1), 175 (2022). 10.1186/s12935-022-02600-3 PMC9063187 35501869

[18] Hamilton TC, Young RC, McKoy WM Characterization of a human ovarian carcinoma cell line (NIH:OVCAR-3) with androgen and estrogen receptors. Cancer Res. 43(11), 5379–5389 (1983). 6604576

[19] Pejovic T, Heim S, Mandahl N Chromosome Aberrations in 35 Primary Ovarian Carcinomas. Genes, Chromosomes & Cancer 4, 58–68 (1992). 1377010 10.1002/gcc.2870040108

[20] Kobel M, Ronnett BM, Singh N, Soslow RA, Gilks CB, McCluggage WG. Interpretation of P53 Immunohistochemistry in Endometrial Carcinomas: Toward Increased Reproducibility. Int. J. Gynecol. Pathol. 38(Suppl. 1), S123–S131 (2019). 29517499 10.1097/PGP.0000000000000488 PMC6127005

[21] Domcke S, Sinha R, Levine DA, Sander C, Schultz N. Evaluating cell lines as tumour models by comparison of genomic profiles. Nat. Commun. 4, 2126 (2013). 23839242 10.1038/ncomms3126 PMC3715866

[22] Surowiak P, Materna V, Maciejczyk A CD46 expression is indicative of shorter revival-free survival for ovarian cancer patients. Anticancer Res. 26(6C), 4943–4948 (2006). 17214367

